# Pruritus in Female Patients

**DOI:** 10.1155/2014/541867

**Published:** 2014-03-10

**Authors:** Julien Lambert

**Affiliations:** Department of Dermatology and Venereology, University Hospital Antwerp/University of Antwerp, Wilrijkstraat 10, 2650 Edegem, Belgium

## Abstract

Pruritus is a frequent symptom in many dermatological diseases. In this review we want to focus on not only itch problems specific to women, namely, pruritic vulvodermatoses, but also the specific pruritic dermatoses of pregnancy. The specific characteristics of the vulva and the hormonal changes during the different age periods make these dermatoses very particular. It seems that vulvar diseases are still underdiagnosed and undertreated. Pruritic vulvar diseases have a huge impact on quality of life. The most common pruritic diseases will be discussed, such as atopic and contact dermatitis, psoriasis, lichen sclerosis, lichen planus, and infectious vulvaginitis. We focus on the diagnostic issue of these diseases and will consider the general principles of therapy.

## 1. Introduction

Pruritus is a frequent symptom in many dermatological diseases. In this review we want to focus on not only itch problems specific to women, namely, pruritic inflammatory vulvar dermatoses, but also the specific dermatoses of pregnancy. Considering these dermatoses we have to take into account the following points: the distinct epithelial characteristics of the vulva in its different regions, the temporal hormonal shifts that lead to cyclic changes in the skin's basic composition, and finally the presence of estrogens receptors on keratinocytes. The changing level of estrogens leads to changes in hydration, collagen content, and concentration of glycosaminoglycans. In addition there will be also changes in vulvovaginal pH and microflora compositions [1].

Vulvovaginal pH is high in childhood, but in puberty the pH starts to decrease from an average of 7 to an average of 4 in adult women.* Lactobacilli* start to colonize the vulvovaginal area. In the first half of the hormonal cycle estrogen levels rise and vulvovaginal epithelial cells proliferate. In the second half of the cycle, which is progesterone mediated, the keratinocytes desquamate. There are also changes of the bacterial flora during the hormonal cycle. Also the pH levels are going to fluctuate and eventually cause pruritus; an increase of pH may activate the proteinase-activated receptor-2 (PAR-2) which is a well-known itch mediator. Due to the decrease of estrogens, vaginal pH is going to rise in menopause [1].

## 2. Atopic and Contact Dermatitis

The commonest vulvar dermatosis in both adults and children is dermatitis. The majority of these patients are atopic [2]. In prepubertal girls atopic and irritant dermatitis occurs often together. Clinical examination shows erythematous and scaly labia majora with frequently rugosity, due to lichenification. The labia minora may be erythematous and scaly. The itch is constant, and the dermatosis is fluctuating. Irritant contact dermatitis may be due to poor hygiene habits or excess use of soap or prolonged wearing of wet swimming suits. Allergic contact dermatitis is very unusual in children because the exposition to potential allergens is low [2].

**Figure 1:**
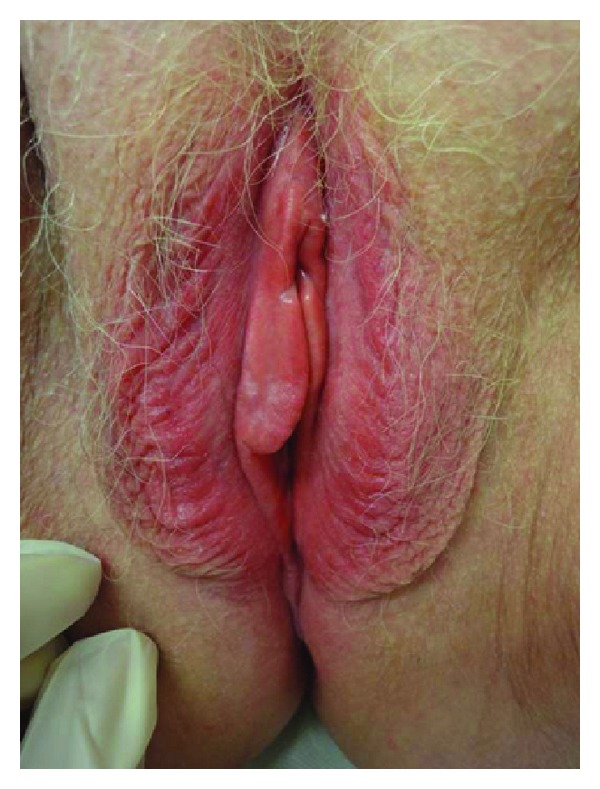
Allergic contact dermatitis to an intimate product.

In adult women allergic and irritant contact dermatitis accounts for around 50% of cases of chronic vulvovaginal pruritus [3]. It may complicate the presentation of other dermatoses. Theoretically there is an increased risk for sensitization: because of differences in structure, occlusion, and hydration and susceptibility to friction, vulval skin is more permeable than exposed skin [4]. The predominant symptom is itch, but burning and pain may also be present, especially if fissures occur. Clinical examination shows erythema and swelling and in chronic cases lichenification is frequently present (Figure 1). Common irritants include soaps, antiseptics, lubricants, spermicides, tampons, sanitary pads, and synthetic underwear. Several studies have highlighted the usefulness of patch testing in case of vulval pruritus [4–6]. A prospective study showed a very high rate of contact sensitivity in patients presenting with vulval pruritus. One or more clinically relevant allergens were found in 44% of the subjects tested [5]. Many relevant allergens did not belong to the European standard series so there is a need for extended patch testing. Interesting to note is that a study demonstrated that 47% of patients with lichen sclerosus had positive patch tests [7]. Topical anesthetics and antibiotics, preservatives, dyes, and perfumes are potential allergens. In order to get a complete list of all the topical applications that women use, do take into account that women regularly use preparations available over the counter. Excessive cleansing of the vulvar skin, as well as urinary and fecal incontinence, may also precipitate to an irritant dermatitis. Finally estrogen-deficient patients are particularly prone to irritant contact dermatitis [8]. Management consists in the removal of all irritants and potential allergens and application of topical steroids until the skin returns to normal.

## 3. Lichen Sclerosus

**Figure 2:**
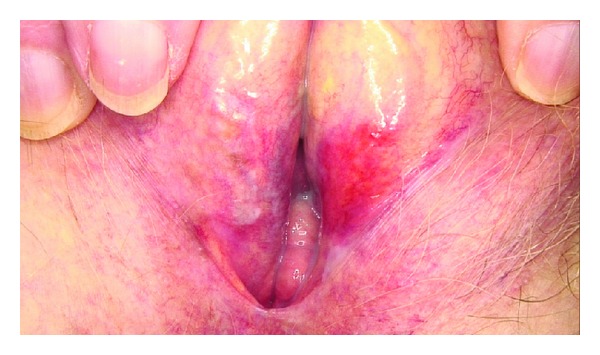
Lichen sclerosus in a postmenopausal woman.

Lichen sclerosus (LS) is a chronic inflammatory dermatosis of unknown etiology first described by Hallopeau in 1897 as an atrophic form of lichen planus [9]. Most cases are seen in prepubertal girls or in postmenopausal women. A possible association with psoriasis has been suggested [10]. The classical presentation is a “figure- of-eight” shaped white plaque around the vulva and anus. Classically it is taught that LS does not affect the vagina, in contrast to lichen planus, which is an important clue in the differential diagnosis. A few cases of LS with vaginal involvement have been reported [9]. Atrophy, erosions, fissures, and ecchymoses may also be present. In advanced cases a loss of genital architecture may occur with subsequent effacement of the labia minora and clitoris (Figure 2). In most cases the itch is predominant but some women will complain more of soreness, burning, and pain. Some pediatric cases resolve with puberty, while others may continue to adulthood. The authors who presented very recently the 2 new cases of LS with vaginal involvement put the question forward if this is not underdiagnosed because the vagina may not be examined carefully for LS or because lesions may be subtle or atypical. Both presented cases had significant pelvic organ prolapse and so the vaginal mucosa was more chronically exposed. This brings into question whether squamatization of the vaginal mucosa may play a role in the development of the vaginal LS lesions [9]. The risk of developing squamous cell carcinoma in longstanding cases of lichen sclerosus is 5% or less. Treatment consists of high potency topical corticosteroids, also in younger patients; however, it is proposed to use not the most potent preparations in these younger patients.

## 4. Lichen Planus

The prevalence of lichen planus (LP) in the genital area is much lower than lichen sclerosus [11]. Differential diagnosis with lichen sclerosus is not always easy. Lichen sclerosus is normally confined to the vulva while lichen planus may affect the vulva as well as the vagina. Other localizations such as the scalp, oral mucosa, skin, and nails may help to confirm the diagnosis of lichen planus. Women complain of soreness, itching, burning, and dyspareunia. Three types of vulvar lichen planus have been described: erosive, classical, and hypertrophic [12]. Erosive lichen planus is the far most common variant (85% of the cases). Erosive LP is characterized by erosions involving the introitus, clitoris and clitoral hood, labia minora and majora. A lacy white edge to the erosions is regularly seen. Healing erosions may appear as a glazed erythema. Vaginal involvement is very common and presents with vaginal erythema, contact bleeding, erosions, and scarring with synechiae. In rare cases vaginal lesions may be the only manifestation. Very recently diagnostic criteria for erosive LP of the vulva have been published [13]. The classical type presents with small purple, polygonal papules, with sometimes a reticulate lace pattern. Postinflammatory hyperpigmentation is rather frequent in the flexures. Hyperkeratotic lichen planus presents as single or multiple white-hyperkeratotic papules and plaques. Many patients present with a mix of different clinical subtypes. A very recent study documents that a significant percentage of patients with vulval LP have associated lichen planopilaris [14]. The commonest pattern of scalp lichen planopilaris was that of the frontal fibrosing alopecia variant (FFA). All of these FFA patients also had oral LP. Treatment consists in the first place of topical steroids. Classical LP is normally treated with a moderately potent topical corticosteroid. For hypertrophic disease a very potent topical corticosteroid is indicated. A hypertrophic lesion that responds poorly to treatment requires a biopsy to rule out a malignant lesion. Erosive vulvovaginal lichen planus is difficult-to-treat dermatosis, which is usually chronic and persistent. Systemic therapy has to be taken into consideration if local therapy is insufficient. Because of the rare risk of squamous cell carcinoma, a long term follow-up is necessary.

## 5. Lichen Simplex Chronicus

The clinical presentation of lichen simplex chronicus is also typical on the vulva: the skin is thickened, lichenified, and often hyperpigmented due to chronic rubbing and scratching of the skin. Lichen simplex chronicus may occur secondary to pruritic conditions such as lichen sclerosus or contact dermatitis. It is also important to take neuropathic itch into consideration as etiology of lichen simplex chronicus. This could be associated with sacral spinal compression, postherpetic neuralgia, and diabetic neuropathy [1].

## 6. Psoriasis

Psoriatic lesions on the vulva are more common in children than in adults. There is no difference in the clinical presentation of psoriasis of the vulva in children and adults. In babies it may initially present as napkin psoriasis. Clinical examination shows itchy well-demarcated symmetric red plaques without scaling in the vulvar and perianal regions. The vagina is spared. Genital itch in psoriatic women is very common. A Polish study revealed a high prevalence of vulvar itching and or burning in women with psoriasis. Moreover this vulvar discomfort and accompanying psoriasis had a significant influence on the psychosocial wellbeing of the patients [15]. Treatment is similar in adults and children and consists of moderated-to-potent topical corticosteroids.

## 7. Infectious Vulvovaginitis

In prepubertal girls a group A betahemolytic streptococcal infection can cause vulvar symptoms. In the acute form there is a sudden onset of an erythematous swollen painful vulva and vagina with a thin mucoid discharge. The subacute form presents as pruritic erythematous patches and plaques in the vulvar and perianal regions [2]. These infections are diagnosed by vaginal and perianal swabs. The origin of the infection is thought to be a pharyngeal infection; however, clinical signs are not always present. Treatment consists of oral penicillin or amoxycilline. Pinworm is a common cause of vulvar and perianal pruritus in children. It may be associated with eczematous lesions and is treated with mebendazole.

Vulvovaginal candidiasis does not occur normally before menarche. On the contrary, many women of reproductive age experience one or more episodes of vulvovaginal candidiasis. Vulvovaginal candida colonization occurs in at least 20% of all women. It is an estrogen dependent process, so it occurs almost exclusively in the reproductive years, especially in the premenstrual period, when hormone levels are high.

Pregnancy, antibiotic use, hormonal contraceptive medication and hormone replacement therapy, and tamoxifen may increase estrogen levels and could be responsible for more frequent colonization and infections [1]. Also changes in the immune system, such as diabetes, HIV, thyroid disease, lupus, and corticosteroid use can cause yeast infections. Not all patients at risk develop Candida infections. Genetic variation plays an important role in host susceptibility. Common polymorphisms in genes of the immune system have been associated with recurrent vulvovaginal candidiasis [16]. Patients complain of itching and burning of the vulva and also a white discharge and vulvovaginal redness. Reliable diagnosis is based on the correlation of clinical features with mycological evidence [17]. In most of the cases, Candida albicans is responsible. An asymptomatic colonization does not need to be treated, except in case of immunosuppression or chronic recurrent vulvovaginal candidiasis [18]. For the treatment of an acute vulvovaginal candidiasis, polyenes, imidazoles, or ciclopiroxolamine in local therapy are proposed or oral triazoles for 1 to 6 days [18]. In case of chronic recurrent* C. albicans* vulvovaginitis the best results are obtained with an individualized decreasing-dose maintenance fluconazole regimen [19]. A German recommendation proposes a prophylactic local treatment of asymptomatic vaginal candida colonization during the last 6 weeks of pregnancy to protect the baby during vaginal delivery. A significant reduction of neonatal candida infection rates was observed [18]. Other species such as* C. glabrata*,* C. tropicalis*, and* C. parapsilosis* may sometimes occur and they are much more difficult to treat.

## 8. Pruritus in Pregnancy

Pruritus is the main dermatological symptom in pregnancy, which is a very particular hormonal period in a woman's life. Pruritus is also a very prominent symptom of the specific dermatoses of pregnancy.

The most recent classification includes pemphigoid gestationis, polymorphic eruption of pregnancy, intrahepatic cholestasis of pregnancy, and the new entity atopic eruption of pregnancy, a new “umbrella” concept comprising atopic dermatitis in pregnancy, prurigo of pregnancy, and pruritic folliculitis of pregnancy [20].

### 8.1. Polymorphic Eruption of Pregnancy

Polymorphic eruption of pregnancy previously known as pruritic urticarial papules and plaques of pregnancy occurs in the latest pregnancy weeks or immediately postpartum. It is associated with primigravida, excessive maternal weight gain, and multiple pregnancies [21]. The pathophysiology is unknown, but a relationship with damage of the collagen fibers due to distension and overstretching of skin is suspected [22]. The clinical examination shows in the beginning urticarial papules and plaques and later on a polymorphous aspect is seen in more than 50% of the patients with vesicular, targetoid, and eczematous lesions. These lesions start within the striae distensae on the abdomen and spread to the buttocks and the proximal extremities. The rash spares very typically the umbilical region. The rash generally resolves within 6 weeks. Recurrences are very rare and are only reported in case of multiple pregnancies. Histopathology is not specific, so normally diagnosis is made by clinical picture and history. Treatment consists of topical corticosteroids with or without antihistamines.

### 8.2. Pemphigoid Gestationis

Pemphigoid gestationis, formally known as herpes gestationis, is a rare bullous autoimmune disease, which normally occurs in the second half of the pregnancy or immediately postpartum. The pathogenesis of this disease is based on the production of circulating immunoglobulin G antibodies that bind to bullous pemphigoid antigen 2 (BP-180) in the hemidesmosomes of the dermoepidermal junction, which results in the damage of the membrane and the production of tense bullae. Clinical examination shows typically tense bullae like in bullous pemphigoid in the neighbourhood of urticarial lesions. The lesions start on the abdomen and do not spare the umbilical region and there is no association with the striae distensae. The lesions may involve the total body, but there is no mucosal involvement. The diagnosis is confirmed by histology and especially direct immunofluorescence which shows a linear C3 along the dermoepidermal junction. Pemphigoid gestationis tends to resolve within weeks to months of delivery. There is a higher risk of premature and small-for-gestational age babies [22]. Treatment consists of antihistamines and systemic corticosteroids.

### 8.3. Intrahepatic Cholestasis of Pregnancy

Intrahepatic cholestasis of pregnancy is a condition that has not always been included in the classifications of pregnancy dermatoses because it is not associated with primary skin lesions.

Patients present secondary skin lesions caused by scratching. It is a hormonally triggered reversible cholestatis, occurring in late pregnancy in genetically predisposed women. The incidence in Europe is much lower than in South-America [23]. The pathogenesis is characterized by an inability to excrete bile salts, causing elevated serum bile acid levels, responsible for pruritus in the mother and influencing negatively the fetal prognosis. There is an increased risk of prematurity, intrapartal fetal distress, and stillbirth.

Patients present a sudden-onset pruritus that starts in the palmoplantar regions but becomes very quickly generalized to the entire body. Due to scratching and rubbing, patients present secondary linear excoriations and prurigo nodularis lesions on the extensor surfaces of the arms and legs. Signs of icterus are seen in approximately 10% of the cases. Diagnosis is made by the rise of serum bile acid levels > 11 *μ*mol/L [22]. Normal levels are 6 *μ*mol/L, but during pregnancy 11 *μ*mol/L is tolerated. Liver function tests are normal in 30% of the cases. The elevation of the serum bile acid levels has a prognostic value; in case of levels of >40 *μ*mol/L, the fetal risk is markedly higher [24]. Treatment consists of ursodeoxycholic acid which reduces serum bile acid levels. This treatment reduces maternal pruritus and also fetal prognosis. Recurrences occur in next pregnancies and in case of oral contraceptive treatment.

### 8.4. Atopic Eruption of Pregnancy

Ambros-Rudolph et al. introduced the new term atopic eruption of pregnancy in 2005 to cover all patients formerly given diagnosis of eczema of pregnancy, prurigo of pregnancy, and pruritic folliculitis of pregnancy [20]. A prospective study on pruritic skin diseases in pregnancy had demonstrated a higher prevalence of atopic eczema. This finding was not taken into consideration in former classifications. They observed a considerable overlap among patients with eczema of pregnancy, prurigo of pregnancy, and pruritic folliculitis, both clinically and histopathologically, so they grouped them within a new disease complex “atopic eruption of pregnancy.” There still exist controversies regarding this terminology [25].

This is the most common pruritic condition in pregnancy noted in almost 50% of the patients. Only 20% of the patients suffered from exacerbation of a pre-existing atopic dermatitis as 80% experienced atopic skin lesions for the first time during their pregnancy [20]. These eczematous lesions could be related to the typical dominance of the Th-2 immunity observed during pregnancy. In order to prevent fetal rejection, normal pregnancy is characterized by a lower Th-1 cytokine production and an enhanced Th-2 cytokine production [26]. Atopic dermatitis is considered to be a Th-2 dominant disease. The Th-2 shift associated with pregnancy may explain the exacerbation of atopic dermatitis during pregnancy. In contrast to the other specific dermatoses of pregnancy the onset occurs in 75% of the cases before the third trimester. The skin lesions can be divided in either eczematous type skin (E-type) changes or prurigo type lesions (P-type) [20]. The eczematous type lesions are located in the classical localizations like the face, the neck, the presternal region, and the flexure sides. The prurigo lesions occur on the extensor surfaces of the extremities. Elevated serum IgE levels are present in 30 to 70% of the cases [22]. Fetal prognosis is unaffected. Recurrences in later pregnancies are to expect. The treatment consists in the first place of topical corticosteroids. In severe cases, systemic corticosteroids, antihistamines, and ultraviolet B phototherapy may be considered.

### 8.5. Treatment during Pregnancy

Treating pruritic diseases in pregnancy remains frequently a challenge. Most of the time topical corticosteroids and antihistamines will be treatment of choice. Little is known about the effects of local corticosteroids on the fetus. A recent European evidence-based guideline suggests the following recommendations [27]. Mild/moderate topical corticosteroids are preferred to more potent corticosteroids. Potent/very potent local corticosteroids should be used as second-line therapy as short as possible and meanwhile appropriate obstetric care should be provided because there is an increased risk of fetal growth restriction. A very recent study showed a significantly increased risk of low birth weight in case that more than 300 g of potent or very potent topical corticosteroids during the entire pregnancy was applied [28]. There are no data available to determine if newer lipophilic topical corticosteroids (mometasone furoate, fluticasone propionate, and methylprednisolone aceponate) are associated with a lower risk of fetal growth restriction. On theoretical grounds they have a more favourable side-effect profile. Systemic corticosteroids have a greater potential for fetotoxicity than local corticosteroids because of a greater bioavailability. They are associated with a reduction in fetal birth weight and an increase in preterm delivery.

There is also a lack of knowledge concerning the use of antihistamines during pregnancy. The older, sedating antihistamines such as dimethindene and clemastine are considered as safe because they are already prescribed for very long time [29]. Regarding the use of hydroxyzine during the first trimester, reports concerning a slight higher risk of malformation [30] and risk of neonatal seizures in case of use in late pregnancy [31] invite cautiousness. The antihistamines of the second generation, such as cetirizine, loratadine, fexofenadine, desloratadine, and levocetirizine provoking low or no sedation, are categorized as medications of which we do not have extensive information about use in humans but animal studies could not show evidence of embryotoxicity or teratogenicity [29]. Loratadine and cetirizine are among the second generation antihistamines the ones best studied. They can be prescribed after the first trimester in case of well-considered indications. Administration just before or after the birth has to be avoided.

## 9. Atrophic Vulvitis

Atrophic vulvitis is a common complaint of postmenopausal women. Estrogens have a proliferative influence on the vulvovaginal epithelium and enhance the circulation and the hydration of the skin and connective tissue [32]. The decrease in estrogens is responsible for a thinner epithelium, a loss of turgor, and a decline of the fat depots of the labia majora. The skin becomes vulnerable and dry and is atrophic, erythematous, and desquamative. Patients complain of itching and a burning sensation. Due to loss of glycogen in the vulvar epithelium the colonization of* lactobacilli* is decreased.* Lactobacilli* produces lactic acid from glycogen and produces so an acid pH [32]. A higher pH creates a favorable environment for pathogenic organisms. The treatment consists in the first place of topical estrogen therapy. The systemic resorption is negligible.

## 10. Conclusion

It is only the last years that there are an increasing number of publications on female specific pruritus. Girls and women may experience during the different age groups a series of pruritic dermatoses as shown in Table 1. These diseases may have a high impact on quality of life. It is therefore of outmost important to recognize them early and to treat them adequately. We have still the impression that until now these specific female itch entities are underdiagnosed. Finally, we focused on inflammatory diseases. Nevertheless, we want to mention briefly that especially in elderly women always malignant lesions have to be taken into consideration in the differential diagnosis of pruritic vulvar diseases.

## Figures and Tables

**Table 1:** Frequent pruritic dermatoses during different age groups.

Prepubertal	Reproductive age	Postmenopausal
Atopic dermatitis	Atopic dermatitis (LF)	
	Allergic contact dermatitis	Allergic contact dermatitis (LF)
Irritant contact dermatitis	Irritant contact dermatitis	Irritant contact dermatitis
Psoriasis	Psoriasis	Psoriasis
Lichen sclerosus	Lichen sclerosus (LF)	Lichen sclerosus
Streptococcal infection	Vulvovaginal candidiasis	
	Lichen simplex	Lichen simplex
		Atrophic vulvovaginitis

LF: less frequent than in the other age groups.
